# Identification, conservation, and expression of tiered pharmacogenes in zebrafish

**DOI:** 10.1371/journal.pone.0273582

**Published:** 2022-08-30

**Authors:** Catherine Demery-Poulos, Joseph M. Chambers

**Affiliations:** Department of Pharmaceutical Sciences, College of Pharmacy, Natural and Health Sciences, Manchester University, Fort Wayne, Indiana, United States; Indiana University-Purdue University Indianapolis, UNITED STATES

## Abstract

The number of adverse drug events in the United States is critically high, with annual rates exceeding 1 million cases over the last nine years. One cause of adverse drug events is the underlying genetic variation that can alter drug responses. Pharmacogenomics is a growing field that seeks to better understand the relationship between a patient’s genetics and drug efficacy. Currently, pharmacogenomics relies largely on human trials, as there is not a well-developed animal model for studying preventative measures and alternative treatments. Here, we analyzed pharmacogene expression at two developmental time points in zebrafish to demonstrate the potential of using this model organism for high-throughput pharmacogenomics research. We found that 76% of tiered human pharmacogenes have a zebrafish ortholog, and of these, many have highly conserved amino acid sequences. Additional gene ontology analysis was used to classify pharmacogenes and identify candidate pathways for future modeling in zebrafish. As precision medicine burgeons, adopting a high-throughput *in vivo* model such as the zebrafish could greatly increase our understanding of the molecular pathology underlying adverse drug events.

## 1. Introduction

Approximately 2 million adverse drug events (ADEs) are reported per year in the United States alone, with severe ADEs representing a leading cause of death [1–3]. The number and severity of ADEs pose a large burden on patient wellbeing and healthcare facilities, as over 5% of hospitalizations are associated with an ADE [1, 4]. The field of pharmacogenomics (PGx) strives to prevent a subset of ADEs by understanding the intricate relationship between human genetics and therapeutics [5]. As a division of precision medicine, PGx aims to direct pharmaceutical approaches based on the genotype of an individual patient [5]. Genes whose protein products influence a drug’s pharmacodynamic or pharmacokinetic fate are termed “pharmacogenes,” which are ranked based on the amount of evidence supporting alterations to drug choice or dosing based on a patient’s genotype [5]. While the precise frequency of ADEs caused by drug-gene interactions is unknown, an estimated 65% of patients are prescribed at least one medication with an established pharmacogenetic association over a 5-year period, with a stark 12% prescribed four or more [6]. To mitigate the incidence of ADEs, adverse drug-gene interactions must be discovered before occurring in patients.

Accordingly, a prominent obstacle in the PGx field is the lack of an *in vivo* model to identify novel relationships and gain further molecular insight into previously identified drug-gene combinations. An aquatic freshwater vertebrate, the zebrafish (*Danio rerio*), provides a powerful model for studying pharmacogenomics in a high-throughput manner. The utility of the zebrafish as a genetic model is illustrated by prior preclinical studies in which the knockdown of candidate genes induced similar disease phenotypes in zebrafish as were observed in humans, confirming the pathological basis of the disorders in question [7, 8]. Notably, rescue experiments following candidate gene knockdown in zebrafish have identified putative therapeutic targets and compounds [7, 8]. Zebrafish are a valuable pharmacological model because they resemble human absorption, distribution, metabolism, and excretion (ADME) for many drug classes and have been shown to predict toxicology with 70-80% accuracy [9–11]. Furthermore, zebrafish share approximately 70% of the human genome while harboring a functional equivalent to over 83% of human disease-causing genes [12]. These advantages, along with well-defined techniques for the genetic modification of zebrafish embryos [13], allow for efficient genetic manipulation in combination with pharmaceutical or chemical treatments.

Yet, the disadvantages of a non-mammalian model system must be acknowledged. Particularly, zebrafish are exothermic and possess anatomical differences from humans, including a lack of limbs, lungs, skin, and mammary glands [14, 15]. While these limitations may preclude the use of zebrafish as a final *in vivo* model for certain disorders, the small size and high fecundity of zebrafish permit statistically significant sample sizes at a considerably lower cost than rodent models, reinforcing the unique potential of this species for high-throughput drug and toxin screening [15–17]. This cost-effectiveness is multiplied when using zebrafish embryos and larvae, which are small enough to fit into multiwell plates for various analyses [15, 17]. Furthermore, zebrafish embryos and larvae are optically transparent and exhibit rapid *ex utero* development, undergoing organogenesis of essential organs such as the kidney, liver, gut, and nervous system all within the first week of life [17].

Here, we present initial evidence to support the zebrafish as a model organism to study PGx. We investigated whether Tier 1 and 2 pharmacogenes are expressed in zebrafish at two key developmental time points and calculated the amino acid conservation of these genes between species. Specifically, we identified zebrafish orthologs for a majority of Tier 1 and Tier 2 human pharmacogenes and verified the expression of such orthologs at 3- and 5-days post fertilization (dpf). Amino acid sequence conservation was used in combination with gene ontology (GO) annotation to identify prominent areas for future research. Together, our results indicate that numerous drug-gene interactions can be explored in zebrafish. This system can be further used to evaluate and develop alternative treatments for adverse drug-gene combinations, with the goal of furthering precision medicine.

## 2. Materials and Methods

### 2.1 Zebrafish husbandry and care

Zebrafish were maintained in the Center for Zebrafish Research at the University of Notre Dame. All aspects of the experiments using living zebrafish were performed at the University of Notre Dame with approval from the University of Notre Dame Institutional Animal Care and Use Committee (IACUC) under protocol number 20-09-6240. Wild type (WT) Tübingen strain zebrafish were raised, staged, and anesthetized (0.02% tricaine) as previously described [18, 19].

### 2.2 Ortholog ID and conservation

Human pharmacogenes were identified from the tiering system used by PharmGKB [20, 21]. Publicly available data from CPIC [22] and the FDA [23] were used to compare tiering systems. Genetic orthologs were identified using ZFIN [24], where orthologs are listed in the description as orthologous to the listed human pharmacogene. This information was further supported by the Alliance of Genome Resources [25], where the majority of algorithms indicated the selected genes were orthologs. If a human pharmacogene had multiple zebrafish genes with ortholog potential, according to ZFIN and the Alliance of Genome Resource, we completed analysis of amino acid sequence conservation to determine which gene to list as the supported ortholog for our study. Conservation was determined using the National Center for Biotechnology Information Basic Local Alignment Search Tool [26, 27] to compare the human and zebrafish amino acid sequences gathered from Ensembl [28]. To account for the genome duplication in zebrafish, we also found and listed paralogs for the zebrafish orthologs of each the human pharmacogenes (**S1 Fig**).

### 2.3 Expression data

Previous expression data were accessed using the publicly available resource, ZFIN [24]. We excluded genes from our own expression analysis only if expression was previously indicated for both time points of interest (3- and 5-dpf). Papers that were not available in full text were excluded from our lists because we could not confirm experimental results. RT-PCR was completed as previously published [29]. In summary, three discrete sets of n = 25 zebrafish specimens (i.e., three biological replicates) were collected for each time point (3- and 5-dpf). RNA was extracted using TRIZOL (Ambion) for each group and then isolated and purified using phenol, chloroform, and ethanol. cDNA was synthesized using qScript cDNA SuperMix (QuantaBio cat# 95047), per manufacturer’s instructions. Briefly, cDNA SuperMix was combined with 1 μg RNA template and nuclease free water for a 20 µL final reaction volume. This mixture was incubated in a thermocycler for 5 minutes at 25°C, 30 minutes at 42°C, and 5 minutes at 85°C. Next, standard PCR was performed on the synthesized cDNA using the primers listed (Thermo Fisher, USA) in **S1 Table** with Taq Polymerase and dNTPs (NEB cat# M0273 and N0447, respectively), per manufacturer instructions. Thermocycler conditions included an annealing temperature of 56°C and an extension time of 30 seconds for 35 cycles. Resulting PCR products were analyzed by gel electrophoresis (using 1% agarose gel). For each biological replicate, PCR and gel electrophoresis were performed in triplicate, thus each gene analyzed was completed in biological and technical triplicate. Amplified PCR products indicative of gene expression were confirmed via sequencing by the Genomics CORE Facility at the University of Notre Dame (Indiana, USA).

### 2.4 Gene ontology

For gene ontology (GO) analysis, the categories were chosen from those listed on ZFIN [24]. Each zebrafish gene was analyzed for each category: Biological Process, Molecular Function, and Cellular Process. GO analysis is largely based on algorithms that predict the attributes of gene products in addition to curated results from various publications. The data and conclusions drawn herein are only representative of the tiered pharmacogenes used in this study, and not necessarily supportive of claims for the entire zebrafish genome.

#### 2.4.1 Statistical analysis

We completed statistical tests to validate our findings where appropriate. The Chi-square test of independence was used to compare the number of Tier 1 and Tier 2 orthologs found and the number of expressed vs. not expressed genes. A Student’s T-test was used to compare the average amino acid conservation between Tier 1 and Tier 2 pharmacogenes. GraphPad Prism 7 software was used to complete the statistical analyses. A p-value < 0.05 was considered significant for all tests.

## 3. Results

### 3.1 Identification and amino acid conservation of zebrafish pharmacogene orthologs

**Fig 1:**
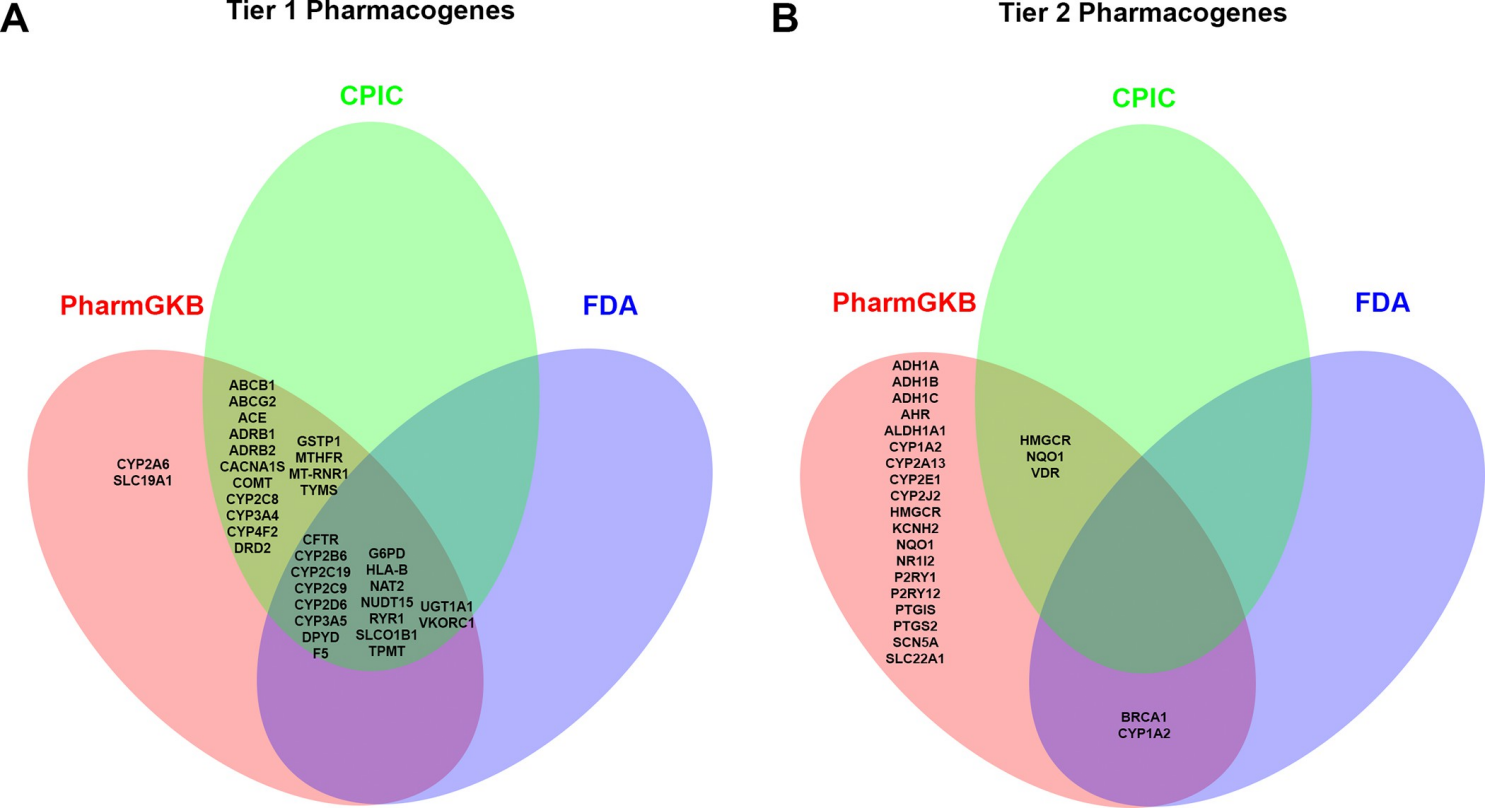
Comparison of tiered pharmacogene sources. Venn diagrams of Tier 1 (A) and Tier 2 (B) pharmacogenes included from the three sources: PharmGKB (red), CPIC (green), and the FDA (blue).

**Fig 2:**
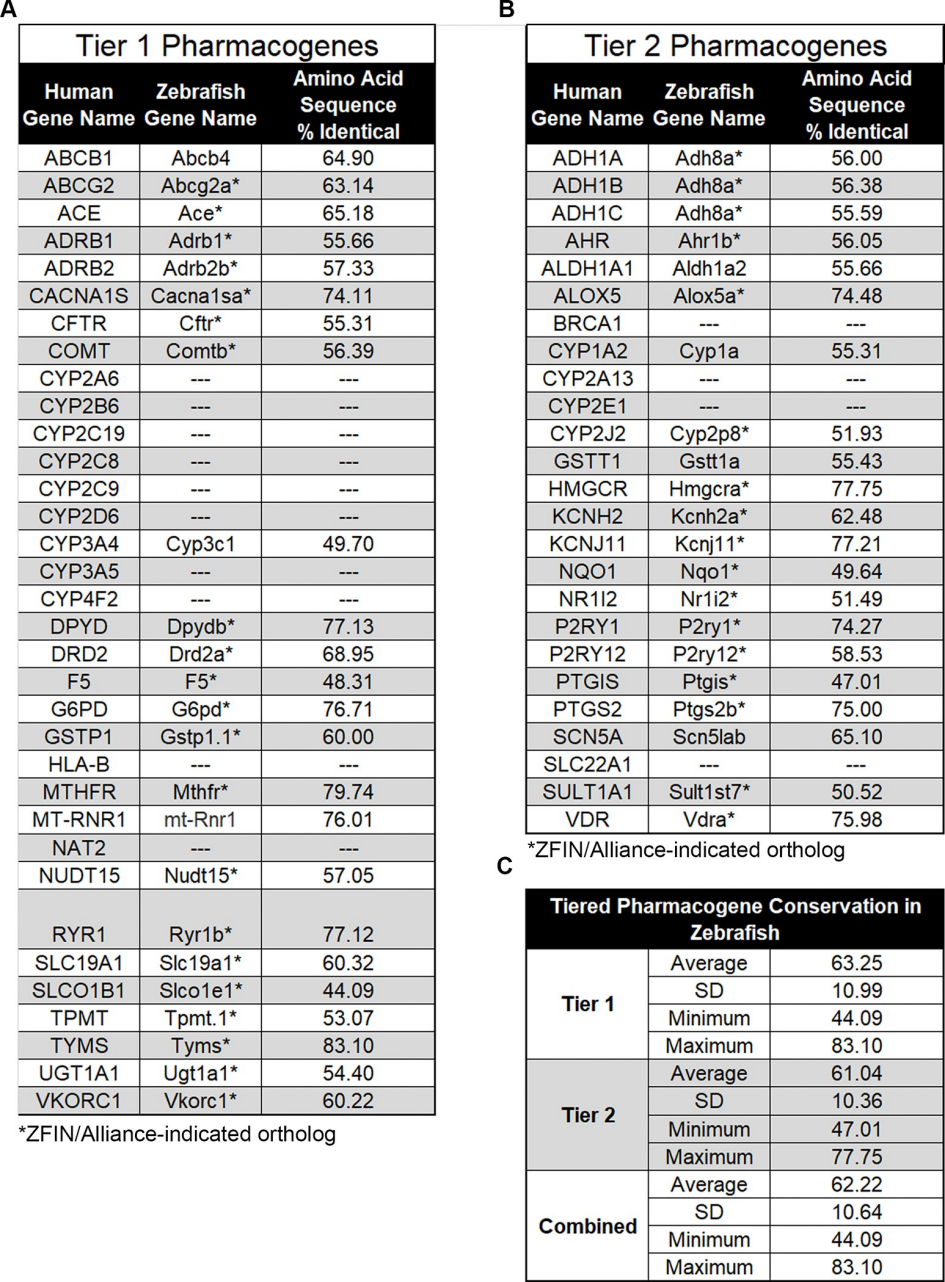
Pharmacogene orthologs and amino acid conservation. Tables with Tier 1 (A) and Tier 2 (B) human pharmacogenes (left), the orthologous zebrafish gene (middle), and the conservation in amino acid sequence as determined by NCBI BLAST. C. Summary tables of averages, standard deviation (SD), minimum, and maximums for Tier 1, Tier 2, and combined. *indicates ZFIN/Alliance-indicated ortholog.

The first step in building the foundation of supporting evidence for zebrafish as a model organism to study pharmacogenomics was to ensure there were a sufficient number of orthologous genes present within the zebrafish genome. It is important to note that the zebrafish genome underwent a duplication event that resulted in a number of paralogs [30]. These paralogs are listed in **S1 Fig**. Our procedure to determine which genes are termed orthologs is described in the Materials and Methods section. However, many of these orthologs are predicted based on amino acid sequence conservation and would need functional assays to further support the claim of orthologous roles. Knockdown of multiple paralogs may be necessary to achieve complete loss of function in the gene of interest. However, that is beyond the scope of the current study. Herein, we compiled a list of Tier 1 and Tier 2 pharmacogenes using available resources such as the Clinical Pharmacogenetics Implementation Consortium (CPIC) [22], PharmGKB [20, 21], and the United States Food and Drug Administration (FDA) [23]; although these sources rank pharmacogenes using different systems, many Tier 1 and Tier 2 genes overlap (**Fig 1**). Next, we used data from the publicly available data repository ZFIN [24] to identify zebrafish orthologs of human pharmacogenes. We found functional orthologs for 24 out of 34 (71%) Tier 1 human pharmacogenes (**Fig 2A**) and 21 out of 25 (84%) Tier 2 human pharmacogenes (**Fig 2B**). There was no statistically significant difference between the number of orthologs identified in Tier 1 compared to Tier 2 pharmacogenes (Chi-square test of independence; p = 0.23). Among the 10 Tier 1 pharmacogenes not found in zebrafish, 8 were members of the Cytochrome P450 (CYP) family of polymorphic enzymes, 1 was Human Leukocyte Antigen (HLA), and the last was N-acetyltransferase 2 (NAT2). The Tier 2 pharmacogenes lacking a zebrafish ortholog included two members of the Cyp-family, Breast Cancer Type 1 (BRCA1), and the solute transporter SLC22A1.

Next, we determined the conservation in amino acid sequences of each zebrafish gene compared to its respective human ortholog; example sequence alignments are shown in **S2 & S3 Figs**. The average amino acid conservation (% identical) for Tier 1 orthologs was 63% +/- 11 (SD), ranging from 44-83% (**Fig 2A**). For Tier 2 orthologs, the average amino acid conservation was 61% +/- 10% (SD) with a range of 47-78% (**Fig 2B**). Together, Tier 1 and Tier 2 human pharmacogenes have 45 identified orthologs (76%) with an average amino acid sequence conservation of 62% +/- 11% (SD), ranging from 44-83% (**Fig 2C**). There was no statistically significant difference between the average amino acid conservation in Tier 1 compared to Tier 2 pharmacogenes (Student’s T-Test; p = 0.66). These data highlight numerous gene candidates with highly conserved amino acid sequences that could be studied using the zebrafish to gain additional insight into tiered drug-gene relationships.

### 3.2 Zebrafish expression of pharmacogene orthologs

Having identified many orthologs in zebrafish, our next approach was to determine if these genes were expressed at biologically relevant and experimentally significant time points. The choice of 3- and 5-days post fertilization (dpf) time points for gene expression analysis was based on a number of factors, including development of the kidney, liver, intestine, and nervous system [19, 31]. After 3-dpf, zebrafish embryos are termed “larvae,” until reaching juvenile status at 30-dpf [19]. Swimming begins at 3-dpf and food-seeking at 5-dpf, enabling complex behaviors to be assayed while the larvae are still small enough to fit in multiwell plates [15, 32]. Importantly, many studies have demonstrated comparable behavioral patterns and teratogenicity between zebrafish larvae and mammals [33, 34]. Thus, the large number of behavior-based assays validated in zebrafish larvae and their high-throughput potential render this stage ideal for pharmacogenomic analyses [35, 36].

**Fig 3:**
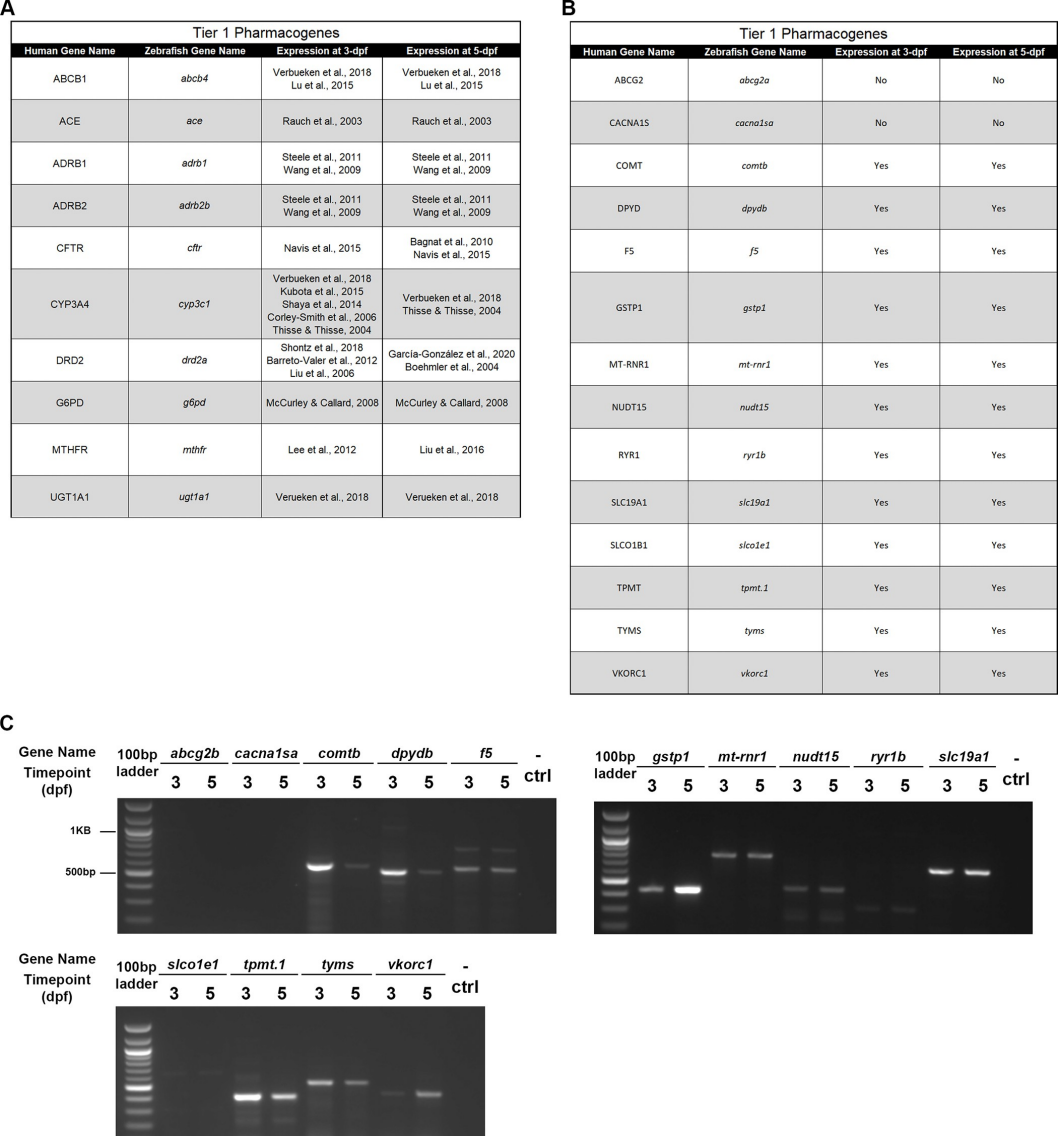
Zebrafish expression of Tier 1 pharmacogenes. A. Previously published literature housing information on expression of Tier 1 pharmacogenes in zebrafish. B. Tier 1 pharmacogene expression in zebrafish: “Yes” indicates expression was noted, “No” indicates no expression found. C. RT-PCR gel results for each Tier 1 zebrafish ortholog not previously identified in the literature. Gene name is listed at the top with the 3-days post fertilization (dpf) and 5-dpf included under the respective gene in alphabetical order. Each gel had a 100-base pair (bp) ladder (left) and a negative control (- ctrl, left).

**Fig 4:**
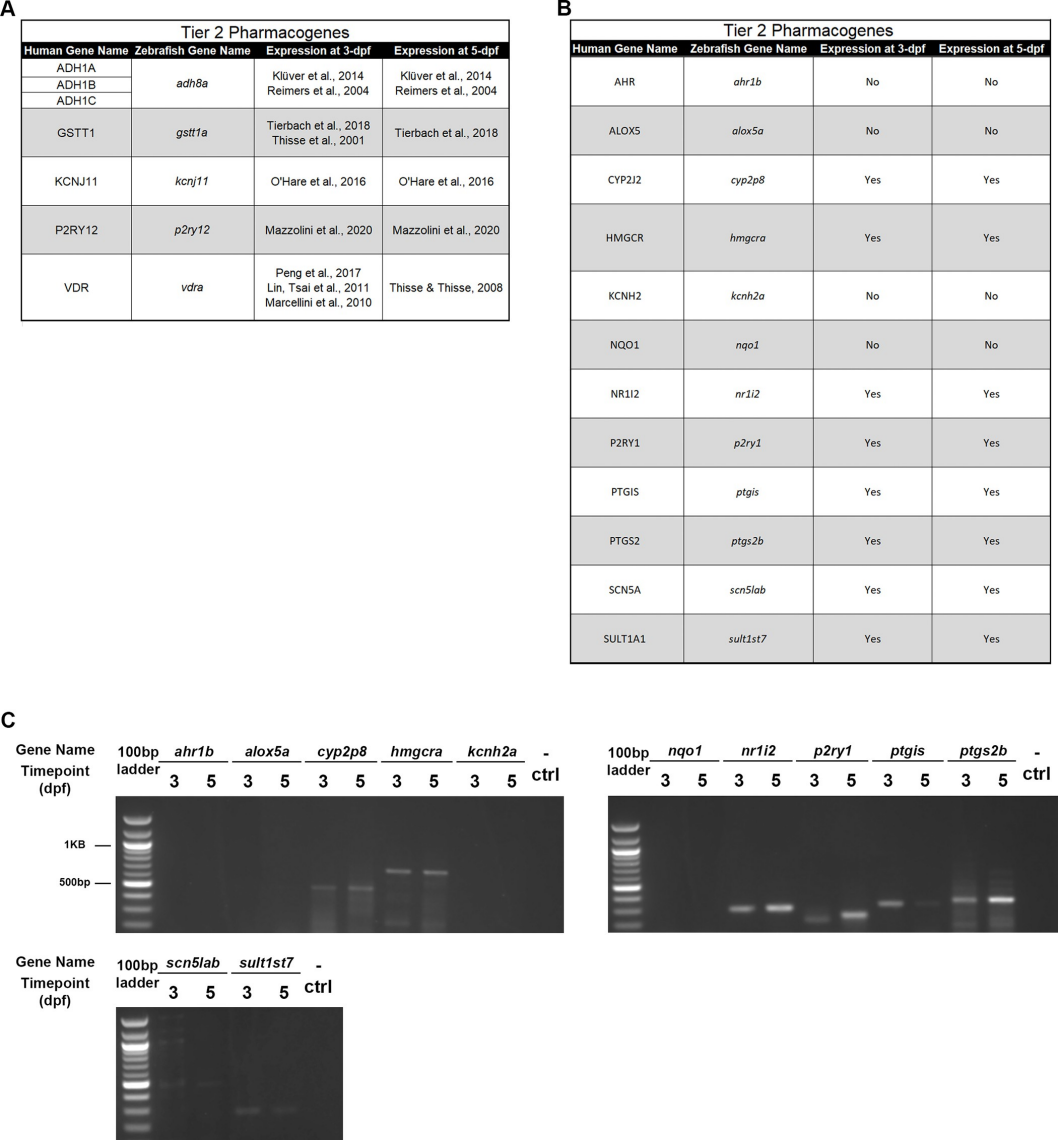
Zebrafish expression of Tier 2 pharmacogenes. A. Previously published literature housing information on expression of Tier 2 pharmacogenes in zebrafish. B. Tier 2 pharmacogene expression in zebrafish: “Yes” indicates expression was noted, “No” indicates no expression found. C. RT-PCR gel results for each Tier 2 zebrafish ortholog not previously identified in the literature. Gene name is listed at the top with the 3-days post fertilization (dpf) and 5-dpf included under the respective gene in alphabetical order. Each gel had a 100-base pair (bp) ladder (left) and a negative control (- ctrl, left).

Expression data were already published for various Tier 1 and 2 genes, which are listed with the appropriate citation by timepoint (3- and 5-dpf) in **Fig 3A** (Tier 1) [12, 37–54] and **Fig 4A** (Tier 2) [55–64]. For the remaining Tier 1 and Tier 2 pharmacogenes without published expression data, we completed RT-PCR with gene-specific primers at both 3- and 5-dpf. All Tier 1 genes, except for *abcg2a* and *cacna1sa*, were shown to be expressed at both 3- and 5-dpf (**Fig 3B and 3C**). In line with this result, we found that all Tier 2 pharmacogenes except for *ahr1b*, *alox5a*, and *kcnh2a* were expressed at both 3- and 5-dpf (**Fig 4B and 4C**). All genes with positive expression results were confirmed via sequencing of the PCR products. There was no statistically significant difference between the number of genes expressed in Tier 1 compared to Tier 2 pharmacogenes (Chi-square test of independence; p = 0.25). Thus, altogether we have evaluated the temporal expression of 26 previously unexplored pharmacogene orthologs and found 20 to be expressed at key timepoints in zebrafish.

### 3.3 Gene Ontology (GO) terms with high conservation in zebrafish

**Fig 5:**
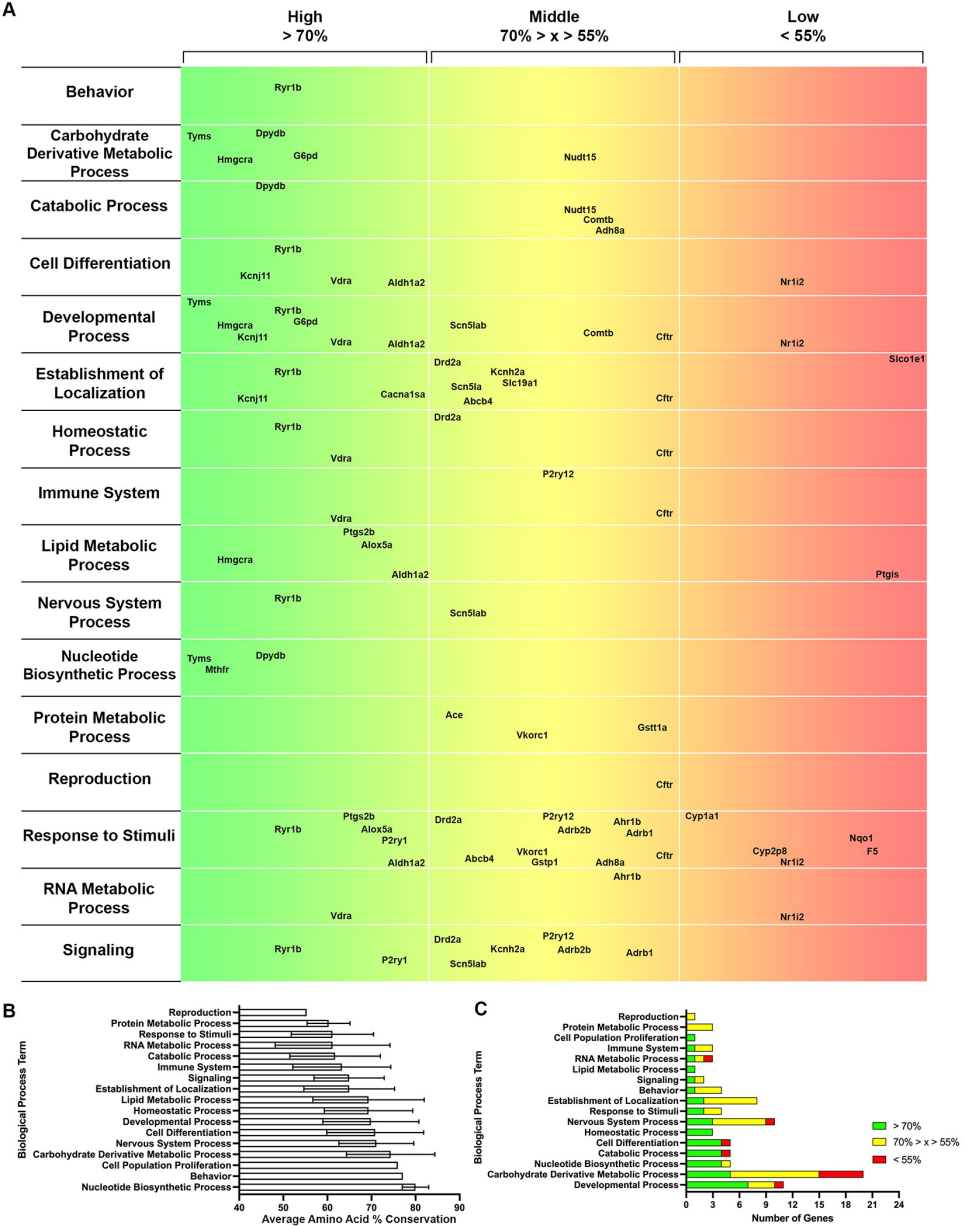
Gene ontology analysis for biological processes of tiered pharmacogenes. A. A map with the Biological Process terms listed (left), amino acid sequence conservation in 3 groups: > 70% = High (green to yellow, left to right), 70% > x > 55% = Middle (yellow to orange, left to right), and < 55% = Low (orange to red, left to right). The genes are placed within the respective horizontal location based on their % conservation. B. Average amino acid conservation for each Biological Process term +/- SD. C. The number of pharmacogenes in each category (High = green, Middle = yellow, Low = red) for each Biological Process term.

**Fig 6:**
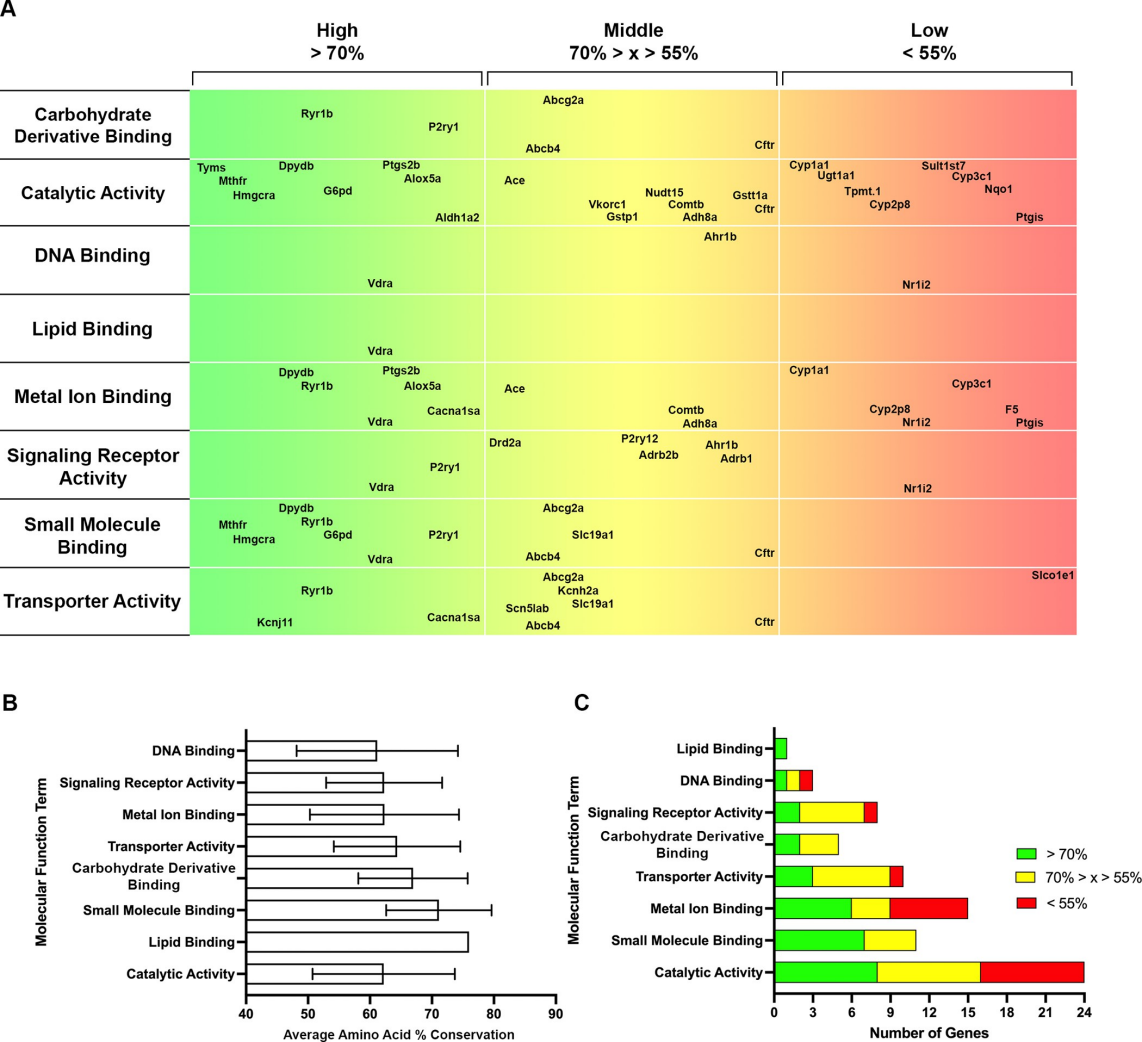
Gene ontology analysis for molecular functions of tiered pharmacogenes. A. A map with the Molecular Function terms listed (left), amino acid sequence conservation in 3 groups: > 70% = High (green to yellow, left to right), 70% > x > 55% = Middle (yellow to orange, left to right), and < 55% = Low (orange to red, left to right). The genes are placed within the respective horizontal location based on their % conservation. B. Average amino acid conservation for each Molecular Function term +/- SD. C. The number of pharmacogenes in each category (High = green, Middle = yellow, Low = red) for each Molecular Function term.

**Fig 7:**
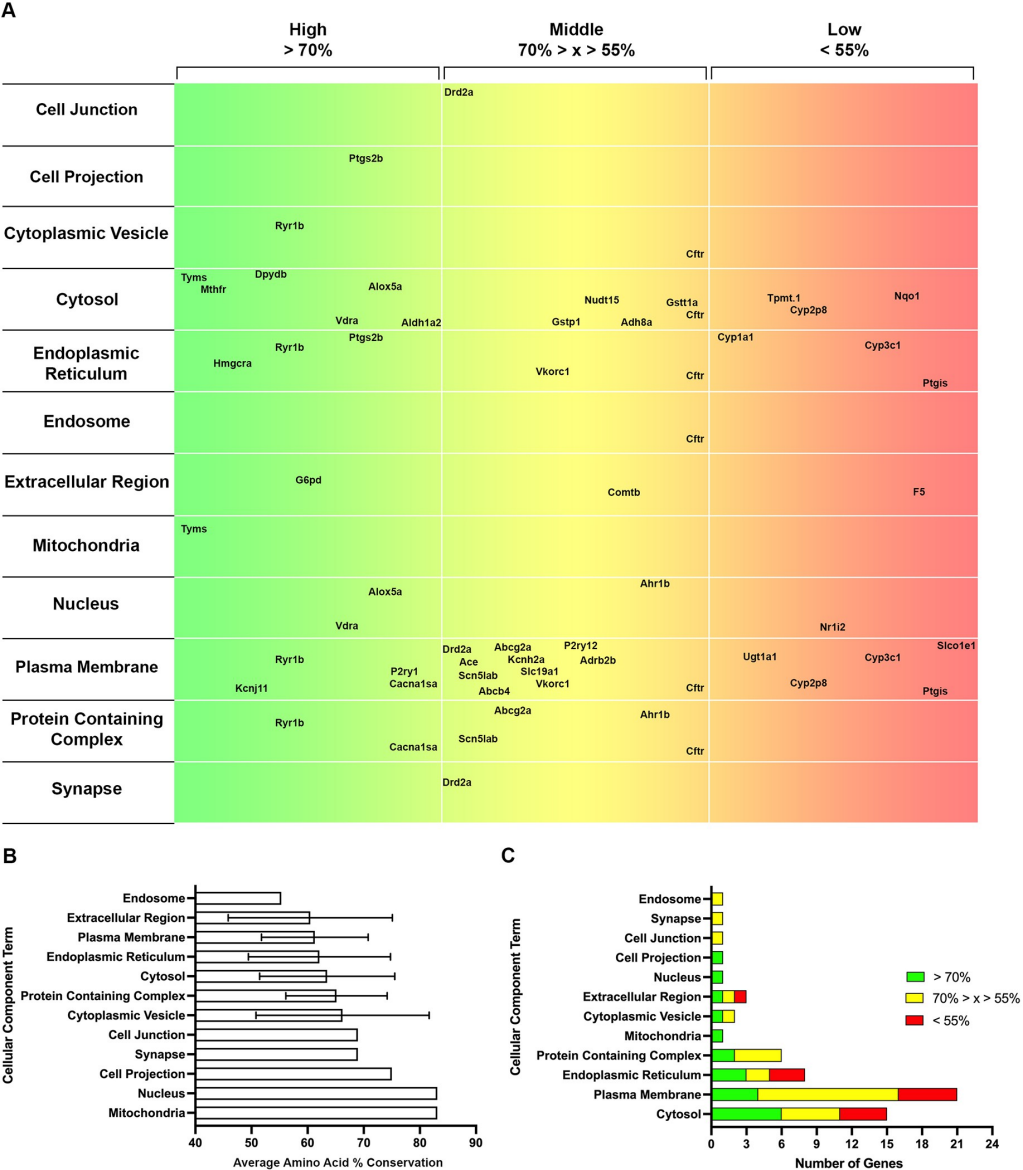
Gene ontology analysis for cellular components of tiered pharmacogenes. A. A map with the Cellular Component terms listed (left), amino acid sequence conservation in 3 groups: > 70% = High (green to yellow, left to right), 70% > x > 55% = Middle (yellow to orange, left to right), and < 55% = Low (orange to red, left to right). The genes are placed within the respective horizontal location based on their % conservation. B. Average amino acid conservation for each Cellular Component term +/- SD. C. The number of pharmacogenes in each category (High = green, Middle = yellow, Low = red) for each Cellular Component term.

Having verified the temporal expression of a majority of Tier 1 and 2 pharmacogene orthologs, we sought to identify systems or processes with high potential for modeling in zebrafish. Gene Ontology (GO) is a bioinformatics approach that categorizes genes by Biological Process, Molecular Function, and Cellular Component. We gathered GO terms for each ortholog identified from ZFIN to discover key terms that housed highly conserved genes. A map illustrating the conservation of amino acid sequences from high (green) to low (red) was completed for Biological Process (**Fig 5A**), Molecular Function (**Fig 6A**), and Cellular Component (**Fig 7A**). These maps list the parent terms for each respective category, and the genes that fall under a given term are located at the appropriate place based on the percent amino acid conservation determined in **Fig 2**. Several genes clustered in the green area indicate a term with multiple highly conserved genes.

To better quantify these clusters, we determined the average of all amino acid sequences for the genes included within a given term (**Figs 5B, 6B and 7B**). To account for the number of conserved genes per term and to mitigate small sample sizes disproportionally affecting category averages, we then ranked the GO terms based on the number of highly conserved genes (> 70% amino acid sequence conservation, green) for Biological Process (**Fig 5C**), Molecular Function (**Fig 6C**), and Cellular Component (**Fig 7C**). This helped identify key Biological Processes, such as Developmental Process, a term that was previously ranked lower based on average amino acid conservation (**Fig 5B**) due to a small number of genes with substantially lower amino acid conservation (*nr1i2* and *ctfr*). Similarly, for the Molecular Function table, Lipid Binding was highly ranked (**Fig 6B**) prior to consideration of the sample size (n = 1). The best-conserved Biological Process terms, defined by the highest number of genes with > 70% amino acid conservation, were Developmental Process, Carbohydrate Derivative Metabolic Process, and Nucleotide Biosynthetic Process (**Fig 5C**). For Molecular Function, the best-conserved terms were Catalytic Activity, Small Molecule Binding, and Metal Ion Binding (**Fig 6C).** Finally, for Cellular Component, the best-conserved terms were Cytosol, Plasma Membrane, and Endoplasmic Reticulum (**Fig 7C**). Altogether, these data indicate key GO terms, such as Developmental Process and Catalytic Activity, are well-conserved in zebrafish and house many pharmacogenes that can be studied to advance human health.

**Fig 8:**
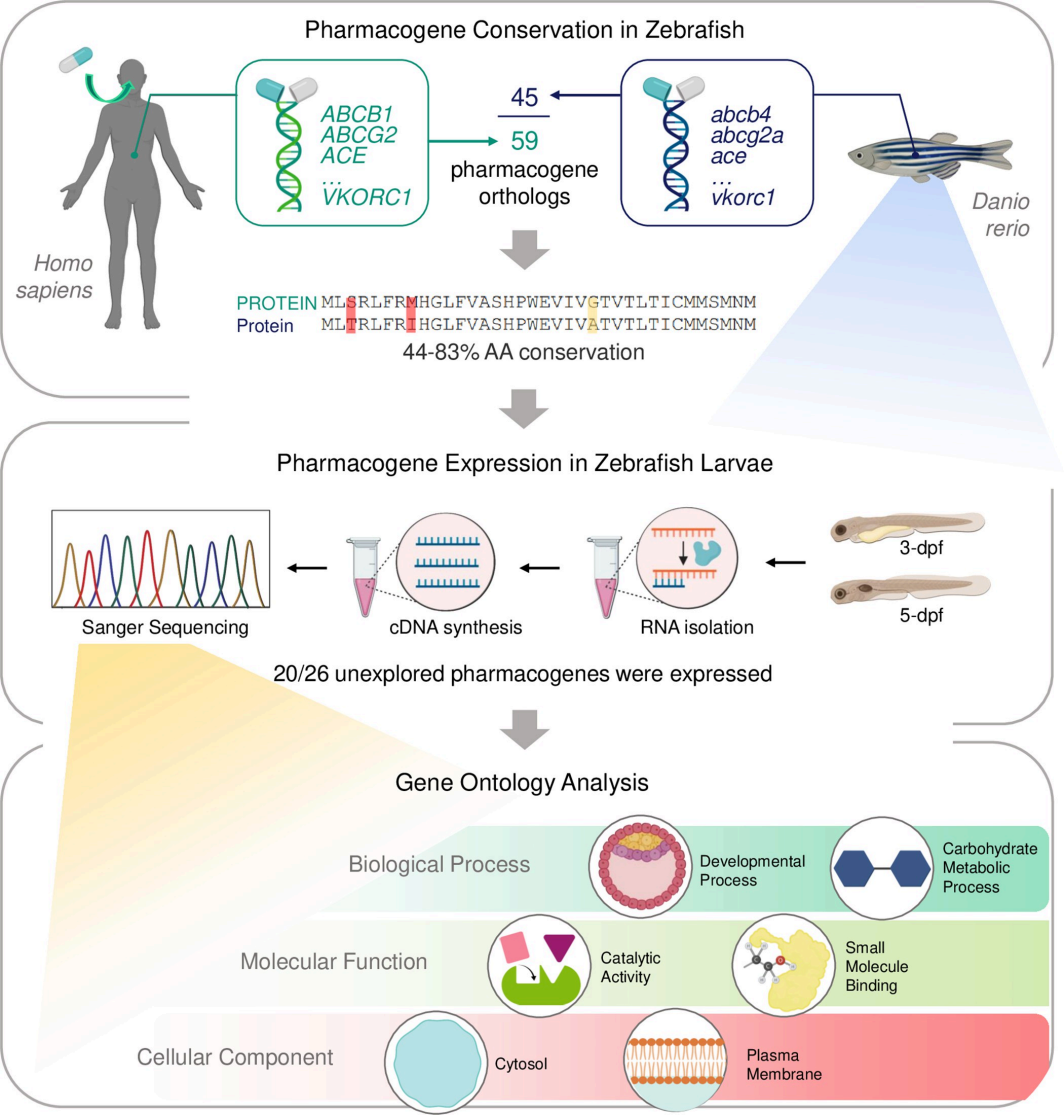
Summary of study design and novel findings. Out of 59 Tier 1 and 2 human pharmacogenes, 45 were determined to have a zebrafish ortholog. The range of amino acid conservation between species was 44 – 83%. For pharmacogenes without published expression data, 3- and 5-dpf zebrafish were collected for gene expression analysis. Expression was demonstrated for 20 out of 26 previously unexplored pharmacogene orthologs. Gene ontology analysis of all 45 pharmacogene orthologs identified the top Biological Process, Molecular Function, and Cellular Component terms for future research, based on the number of highly conserved genes associated with each term.

In summary, we have identified zebrafish orthologs for 45 out of 59 Tier 1 and 2 pharmacogenes, which were found to have a range of 44 – 83% amino acid conservation between species (**Fig 8**). We demonstrated expression at 3- and 5-dpf for 20 out of 26 previously unexplored pharmacogenes in zebrafish (**Fig 8**). Finally, we performed gene ontology analysis on all 45 pharmacogene orthologs to uncover key terms that housed multiple highly conserved genes and may be promising for future pharmacogenomic research in zebrafish (**Fig 8**).

## 4. Discussion

Here, we illustrate the immense potential of zebrafish as a resource for the field of pharmacogenomics research. Starting with 59 combined Tier 1 and Tier 2 pharmacogenes, we have identified 11 genes with high-impact potential for modeling in zebrafish, based on verified expression early in the larval stage and amino acid conservation higher than 70%. Modeling complex human disorders in zebrafish is gaining momentum due to the numerous benefits discussed previously and in various elaborate articles [65–68]. One major benefit is the low cost relative to other *in vivo* models [16, 67, 68], with the cost of screening one drug or compound calculated to be 500x cheaper in zebrafish compared to rats [69]. However, there are drawbacks to modeling human diseases in zebrafish, a non-mammalian species that has undergone a gene duplication event [30]. Critical anatomical and genetic differences exist between the species, which limit the extent that zebrafish can fully recapitulate human disease. Yet, this model is not intended to replace the mouse or rat, but to serve as a high-throughput bridge between *in vitro* analysis and mammalian models [14, 15]. Indeed, complex behaviors such as aggression have been successfully explored in zebrafish using pharmacogenetic and pharmacogenomic models [70]. As always, there is a balance of strengths and weaknesses when deciding which animal model to employ in studying human disease states.

Several examples of modeling human diseases with PGx-relevant genes in zebrafish already exist, albeit with different research goals. For example, the phenotype conferred by the human rs6277 allele of *DRD2* has been successfully modeled in zebrafish using morpholinos against *drd2a* [71], and zebrafish models of neurodegeneration are being employed to identify mechanisms and treatments for Parkinson’s disease [72]. Another gene included in our tiered pharmacogene list that has high sequence conservation and was found to be expressed at 3- and 5-dpf is the gene encoding the mitochondrial 12s rRNA subunit, *mt-rnr1*. Interestingly, variations within *mt-rnr1* are associated with aminoglycoside antibiotic-induced hearing loss [73, 74]. Considering the established use of zebrafish to investigate hearing-related disorders including ciliopathies, this platform could be used to investigate factors contributing to the variable penetrance associated with the A1555G mutation, or the molecular basis for hearing loss associated with the same allele in the absence of aminoglycoside usage [75–77].

In addition to individual genes, we have identified well-conserved Biological Processes (**Fig 5C**) and Molecular Functions (**Fig 6C**) with high-impact potential. Importantly, these categories of high conservation are based on the human pharmacogenes of interest in this study. Thus, these categories are likely different when analyzing the entire zebrafish genome. One of the top identified Biological Processes within the studied genes was Carbohydrate Derivative Metabolic Process, the dysfunction of which is implicated in various disorders. For example, decreased-function HMGCR variants may be associated with new-onset diabetes mellitus [78, 79], illustrating another translational model to explore in zebrafish (Hmgcra; 78% amino acid conservation). Additionally, mutations in glucose-6-phosphate dehydrogenase (G6PD; G6pd; 76% amino acid conservation) are responsible for the most prevalent enzyme deficiency worldwide [80–83]. The FDA has cautioned against the use of several drugs – from anticancer to antimalarial – by G6PD-deficient individuals [84], and targeted modeling in zebrafish could be used to evaluate the safety of alternative treatments for such patients. Furthermore, among the best-conserved Molecular Functions were Catalytic Activity and Small Molecule Binding, which are extremely relevant for exploring pharmacological targets. These are just a few examples of the potential utility gained by modeling well-conserved systems and genes in zebrafish, which also permit larger sample sizes to increase the power of relevant systems.

Despite the conservation of many systems targeted by pharmaceuticals, one clear limitation of pharmacogenomic modeling in zebrafish is the poor conservation of the CYP enzymes, which have a dominant role in human metabolism of xenobiotics [85]. Delineating the relationships between human and zebrafish CYPs is challenging because of teleost genome evolution, including whole genome duplication, uneven gene duplication and loss, further complicated by the presence of multiple Cyp genes per human CYP [86]. While a comprehensive evaluation of Cyp expression in zebrafish has been conducted [86], more studies are needed to determine which aspects of the functional CYP metabolism, including substrate and metabolite specificity, are retained in zebrafish. Alternatively, transgenic zebrafish lines that express human CYP enzymes, such as CYP3A4, have been developed for pharmacokinetic analyses [87]. This concept remains an area for further development within the field.

In this study, we have provided foundational evidence for the use of zebrafish in pharmacogenomic modeling, based on the expression of many Tier 1 and 2 pharmacogenes, including 11 with a high percentage of amino acid conservation (> 70%). Future research should confirm the functionality of these zebrafish pharmacogene orthologs with strong evidence in humans (i.e., Tier 1 and 2) and high amino acid conservation between species, as well as further elucidate the molecular underpinnings of drug-gene pairs. Furthermore, beyond the Tier 1 and 2 pharmacogenes evaluated in this study, CPIC, PharmGKB, and the FDA have identified tens of additional genes, currently untiered, with preliminary data that suggests an association with various drugs that can cause ADEs. In short, we have shown that numerous Tier 1 and 2 pharmacogenes are expressed in the developing zebrafish with high amino acid conservation. We hope this preliminary evidence will encourage further exploration of the zebrafish as a pharmacogenomic model to explore the molecular pathology underlying human ADEs.

## Supporting information

S1 Table Primer information. This lists the forward (FWD) and reverse (RVS) primers that were used for PCR of each target gene listed using zebrafish cDNA.
(TIF)

S1 Fig Paralog information. Lists of Tier 1 Pharmacogenes (Left) and Tier 2 Pharmacogenes (Right) with the corresponding paralogs found on ZFIN. The gene listed as the ortholog was weighted via the Alliance Genome Resource as more closely related to the human Pharmacogene.
(TIF)

S2 Fig Sequence alignment example. Here we provide an example of the most similar (in terms of amino acid sequence) Tier 1 gene (TYMS) to visualize the similarities amongst amino acids over the length of the sequence.
(TIF)

S3 Fig Sequence alignment example. Here we provide an example of the most similar (in terms of amino acid sequence) Tier 2 gene (HMGCR) to visualize the similarities amongst amino acids over the length of the sequence.
(TIF)

S1 Raw image (PDF)
