# Supplementary figures and images for: Identification, conservation, and expression of tiered pharmacogenes in zebrafish
Source: PLoS One. 2022 Aug 30;17(8):e0273582. doi: 10.1371/journal.pone.0273582 (PMC9426904; doi:10.1371/journal.pone.0273582)

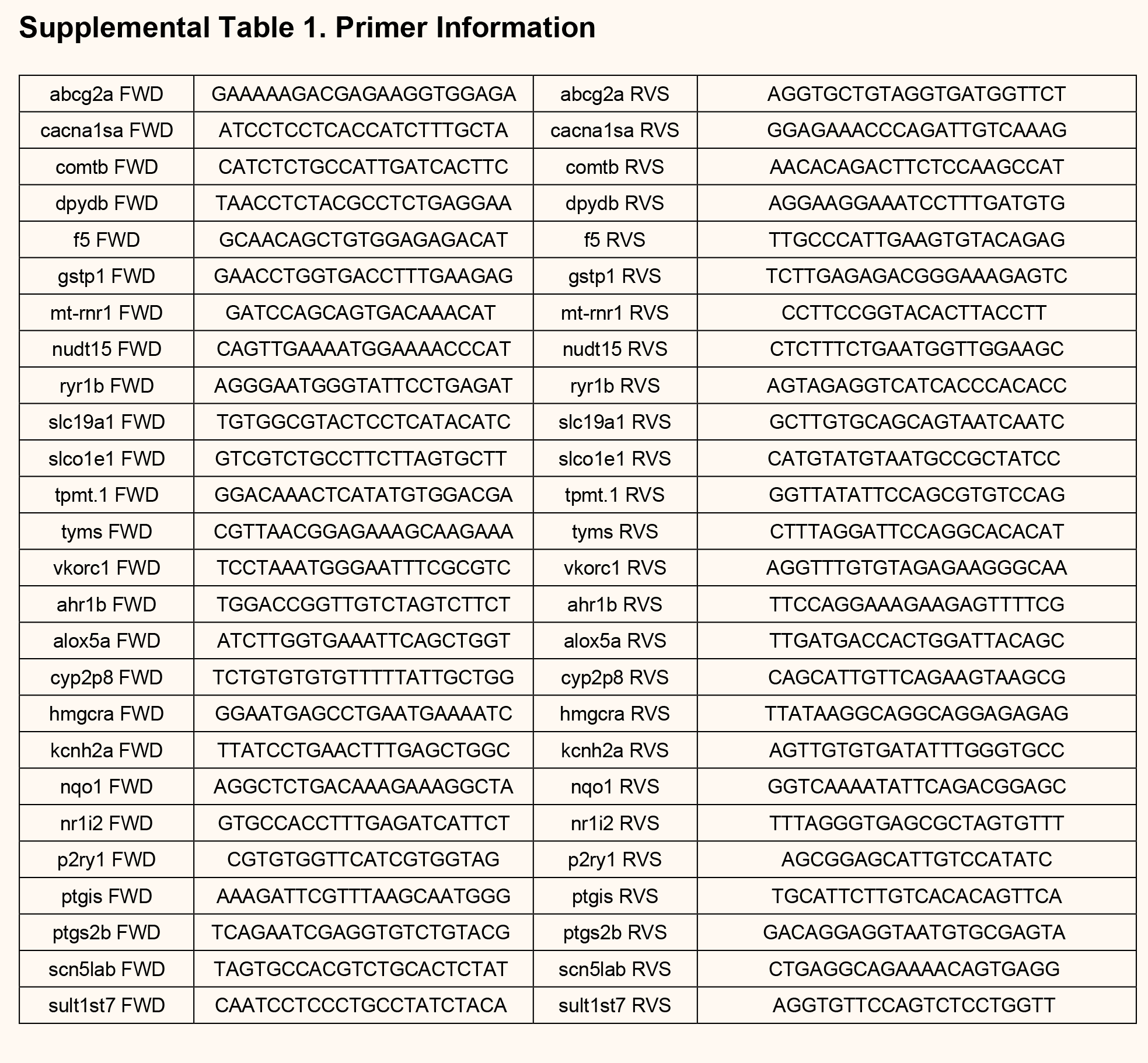

Supplement: S1 Table — This lists the forward (FWD) and reverse (RVS) primers that were used for PCR of each target gene listed using zebrafish cDNA. (TIF) [file pone.0273582.s001.tif]

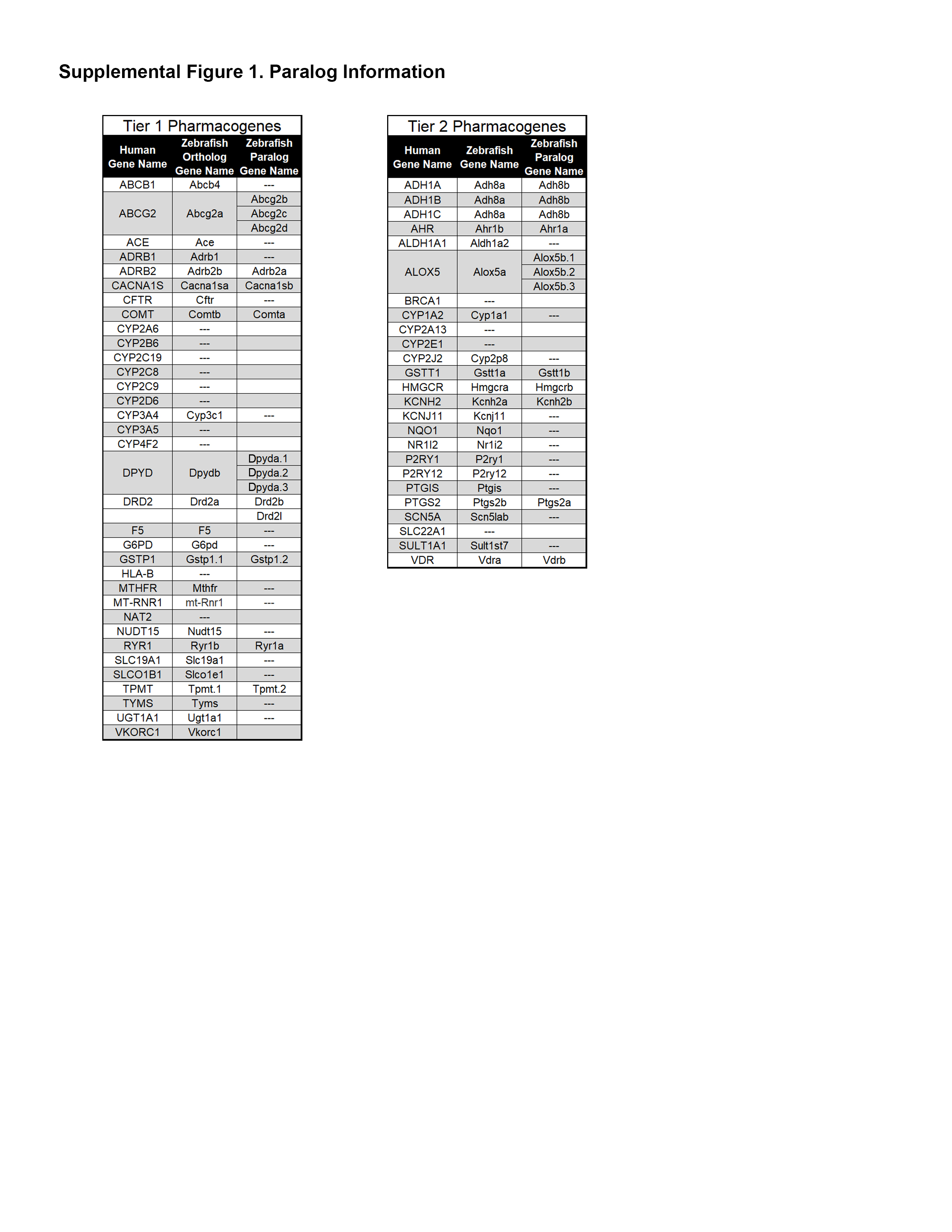

Supplement: S1 Fig — Lists of Tier 1 Pharmacogenes (Left) and Tier 2 Pharmacogenes (Right) with the corresponding paralogs found on ZFIN. The gene listed as the ortholog was weighted via the Alliance Genome Resource as more closely related to the human Pharmacogene. (TIF) [file pone.0273582.s002.tif]

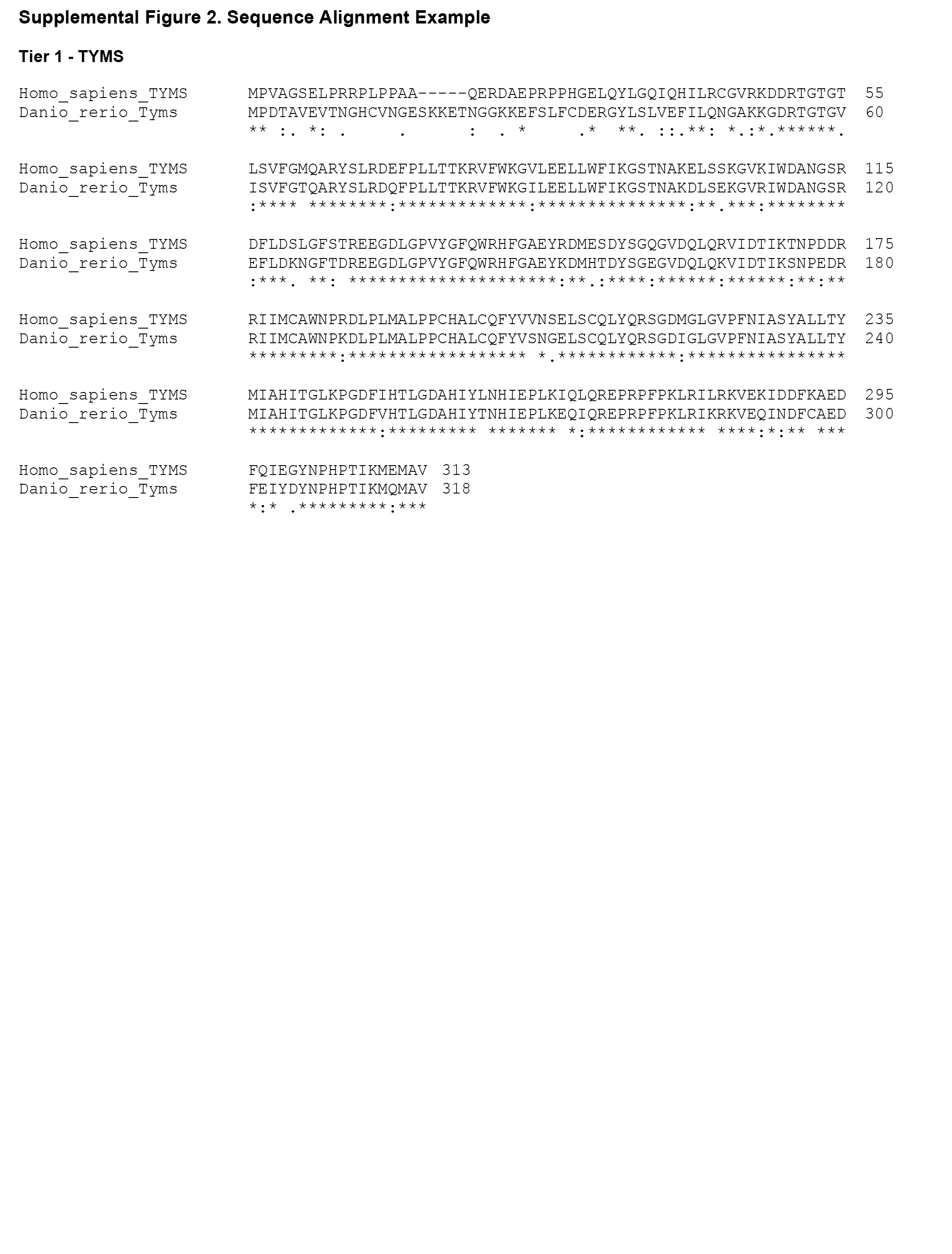

Supplement: S2 Fig — Here we provide an example of the most similar (in terms of amino acid sequence) Tier 1 gene (TYMS) to visualize the similarities amongst amino acids over the length of the sequence. (TIF) [file pone.0273582.s003.tif]

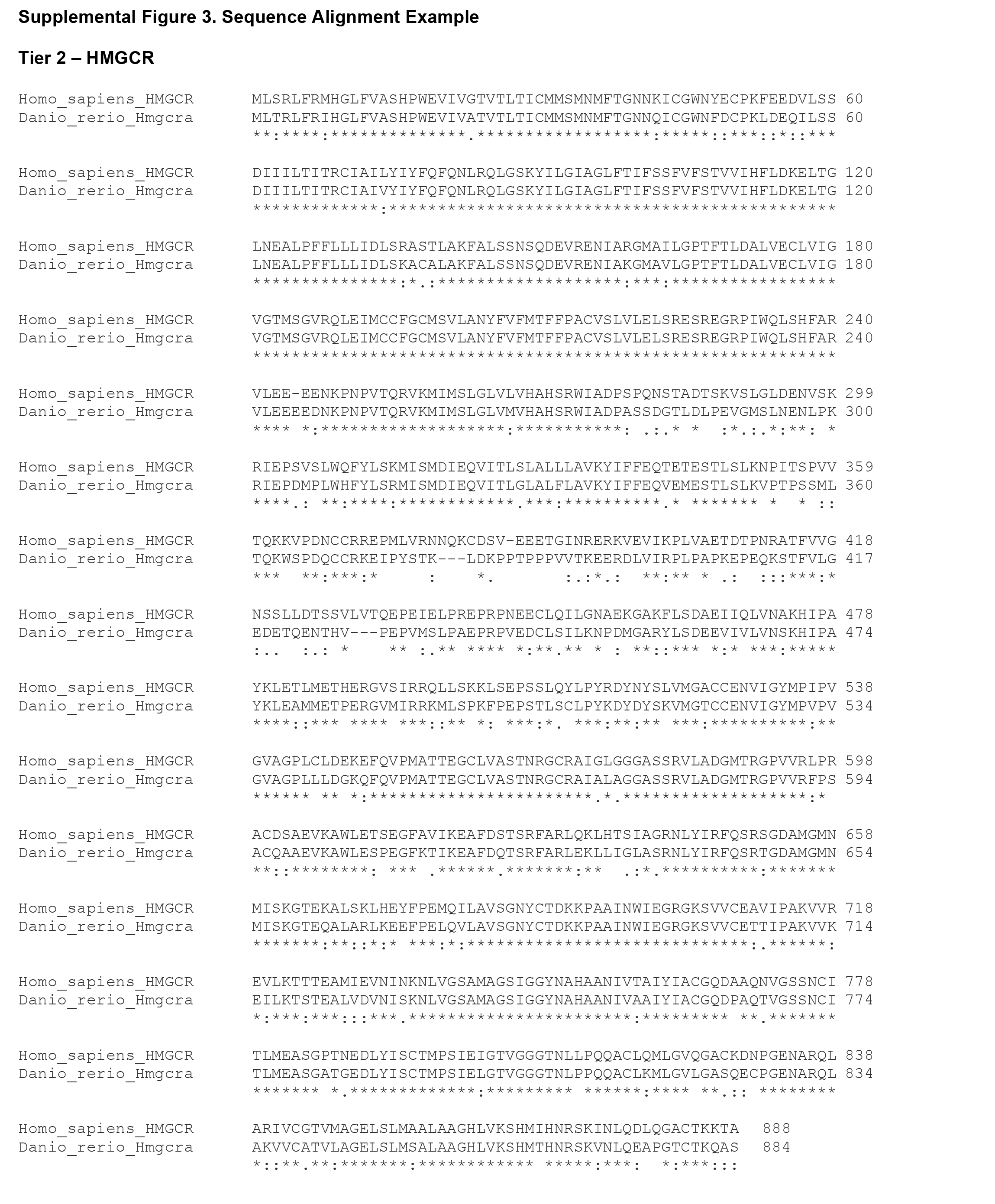

Supplement: S3 Fig — Here we provide an example of the most similar (in terms of amino acid sequence) Tier 2 gene (HMGCR) to visualize the similarities amongst amino acids over the length of the sequence. (TIF) [file pone.0273582.s004.tif]
