# Supplementary material for: Identification, conservation, and expression of tiered pharmacogenes in zebrafish
Source: PLoS One. 2022 Aug 30;17(8):e0273582. doi: 10.1371/journal.pone.0273582 (PMC9426904; doi:10.1371/journal.pone.0273582)

In order of how they appear in this file:

1: Figure 3C gel 1 (beginning with *abcg2b* ending with *f5*),

2: Figure 3C gel 2 (beginning with *gstp1* ending with *slc19a1*),

3: Figure 3C gel 3 (beginning with *slco1e1* ending with *vkorc1*).

4: Figure 4C gel 1 (*ahr1b* – *kcnh2a*),

5: Figure 4C gel 2(*nqo1* – *ptgs2b*),

6: Figure 4C gel 3 (*scn5lab* and *sult1st7*)

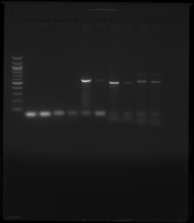

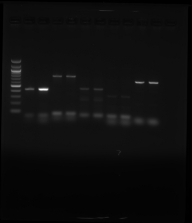

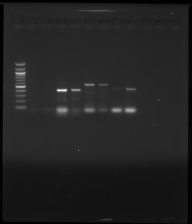

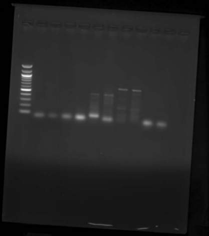

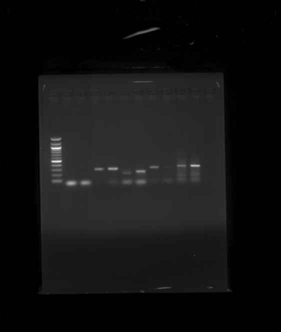

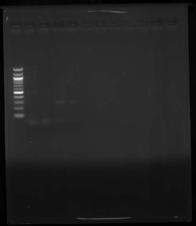

Supplement: S1 Raw image — (PDF) [file pone.0273582.s005.pdf]
